# Changes in Blood Lead Levels Associated with Use of Chloramines in Water Treatment Systems

**DOI:** 10.1289/ehp.9432

**Published:** 2006-11-07

**Authors:** Marie Lynn Miranda, Dohyeong Kim, Andrew P. Hull, Christopher J. Paul, M. Alicia Overstreet Galeano

**Affiliations:** Nicholas School of the Environment and Earth Sciences, Duke University, Durham, North Carolina, USA

**Keywords:** blood lead levels, chloramines, GIS, lead risk, water quality

## Abstract

**Background:**

More municipal water treatment plants are using chloramines as a disinfectant in order to reduce carcinogenic by-products. In some instances, this has coincided with an increase in lead levels in drinking water in those systems. Lead in drinking water can be a significant health risk.

**Objectives:**

We sought to test the potential effect of switching to chloramines for disinfection in water treatment systems on childhood blood lead levels using data from Wayne County, located in the central Coastal Plain of North Carolina.

**Methods:**

We constructed a unified geographic information system (GIS) that links blood lead screening data with age of housing, drinking water source, and census data for 7,270 records. The data were analyzed using both exploratory methods and more formal multivariate techniques.

**Results:**

The analysis indicates that the change to chloramine disinfection may lead to an increase in blood lead levels, the impact of which is progressively mitigated in newer housing.

**Conclusions:**

Introducing chloramines to reduce carcinogenic by-products may increase exposure to lead in drinking water. Our research provides guidance on adjustments in the local childhood lead poisoning prevention program that should accompany changes in water treatment. As similar research is conducted in other areas, and the underlying environmental chemistry is clarified, water treatment strategies can be optimized across the multiple objectives that municipalities face in providing high quality drinking water to local residents.

Exposure to lead has long been recognized as hazardous to human health (Pueschel et al. 1996). Until the 1970s, concerns about lead poisoning primarily focused on acute exposures resulting in convulsions, paralysis, anemia, and gastrointestinal problems (International Program on Chemical Safety 1995). There is now recognition of significant asymptomatic health effects at levels much lower than those previously considered safe, particularly for children (Bellinger 1995; Canfield et al. 2003a, 2003b; Gardella 2001; Huseman et al. 1992; Lanphear et al. 2000; Needleman and Gatsonis 1990; Needleman et al. 1990, 1996; Schwartz 1994; Schwartz et al. 1986).

Environmental lead exposure occurs through ingestion or inhalation of lead particles (Davidson and Rabinowitz 1991). Most childhood lead uptake in the United States results from exposure to deteriorating lead paint in household dust and soil and to lead in soil from historic deposition from mobile sources (Davidson and Rabinowitz 1991; Mielke and Reagan 1998), although drinking water can be a source of chronic exposure (Maas et al. 2005a; Raab et al. 1987; Sherlock et al. 1984; Thomas et al. 1979). Although drinking water is not the primary route of exposure for most children, the U.S. Environmental Protection Agency (EPA 1994) has estimated that 14–20% of total childhood lead exposure in the United States is from drinking water (U.S. EPA 1994).

In 1991, the U.S. EPA (1991) set a maximum contaminant level goal for lead in drinking water of zero and an action level of 15 ppb. Although water supplies themselves can be contaminated with lead, most lead in drinking water comes from residential plumbing (Davidson and Rabinowitz 1991). Lead piping was uncommon after the 1930s, but lead soldering was common and legal until 1986, and some plumbing fixtures today still contain lead (Maas and Patch 2004; Safe Drinking Water Act Amendments of 1986; Troesken and Beeson 2003). Lead is soluble in water, and this solubility is markedly increased by high water softness and acidity (Davidson and Rabinowitz 1991; Gaines 1913; Raab et al. 1993).

Drinking water preparation can differ significantly across water systems, depending on the type and quality of source water, and is intended to protect the public from microbial pathogens, prevent dental caries, reduce harmful disinfection by-products, and reduce metal contamination from pipes (U.S. EPA 1999). To achieve these goals, drinking water treatment systems generally process water using a number of additives, including fluoride, disinfectants (historically primarily chlorine), coagulants to precipitate fine solids, and anti-corrosivity agents to reduce leaching of metals from plumbing into the water (Durham Department of Water Management 2004; Edwards and Dudi 2004; Edwards et al. 1999; Schock 1980, 1989). The corrosivity of water can be highly sensitive to small fluctuations in pH, alkalinity, temperature, oxidation potential, and concentrations of individual chemical species (Edwards and Dudi 2004; Edwards et al. 1999; Schock 1980, 1989; Schock et al. 2001; Vasquez et al. 2006).

Trihalomethanes, by-products of chlorine-based disinfection processes, have long been recognized as carcinogenic, neurotoxic, and teratogenic (Cotruvo 1981; Maxwell et al. 1991). Concerned over the enduring presence of these chlorination by-products, in 1998 the U.S. EPA published its *Stage I Disinfection By-products Rule,* requiring water treatment systems to reduce the formation of these disinfection by-products (U.S. EPA 1998a). Exceedances of the U.S. EPA trihalomethane standard (U.S. EPA 1998a) have led an increasing number of municipal water treatment facilities to switch from chlorine to chloramine use.

Chloramines alter water chemistry and often must be accompanied by other changes to water treatment (U.S. EPA 1999). Several recent studies provided evidence that the introduction of chloramines to water systems with lead-containing pipes, fixtures, or solder may increase the amount of dissolved lead in water because of changes in water chemistry; interactions with additives such as coagulants or fluoridation agents may remove lead dioxide scales originally formed during decades of chlorine-based disinfection (Edwards and Dudi 2004; Maas et al. 2005b; Schock 1990; Schock et al. 2001; Switzer et al. 2006). This leaching might be managed to some extent by the addition of anticorrosivity agents during the water treatment process; however, the details of all the related environmental chemistry are not fully understood and are highly dependent on the particular chemical interactions found in each water treatment and distribution system (Edwards and Dudi 2004; Edwards et al. 1999; Lin et al. 1997; Schock 1989).

In two highly publicized incidents in Washington, DC (Edwards and Dudi 2004; Tiemann 2005), and in Greenville, North Carolina (Renner 2005), tests of residential tap water showed high levels of lead following the introduction of chloramines for disinfection purposes. Water quality monitoring regulations have been established to ensure that water reaching consumers meets all safety standards (Safe Drinking Water Act Amendments of 1986; U.S. EPA 1991, 1998a, 1998b). However, water lead levels can be difficult to measure to adequately characterize human exposure. The location of potential lead sources (pipes vs. fixtures or solder) and timing of sample collection, as well as small changes in water chemistry, can drastically affect observed/measured water lead levels (Edwards and Dudi 2004; Renner 2006; Schock 1990). As a result, routine monitoring in water systems in Washington, DC, and North Carolina failed to detect increased levels of lead directly after changing disinfectants from chlorines to chloramines (Edwards and Dudi 2004; Maas et al. 2005a; Renner 2004, 2005). In Washington, DC, the increase was not detected and reported until almost a year after the change in the disinfection process had occurred because elevated samples were invalidated in 2001. After high lead levels were detected in water in early 2002, sampling protocols were altered in 2003. This made an informed assessment of human exposure to increased lead levels difficult to undertake, because test results can be heavily affected by changes in sampling methods (Edwards and Dudi 2004; Schock 1990).

Although some documentation of changes in water lead levels exists, only one published study, focused on Washington, DC, has evaluated blood lead levels (BLLs) associated with changes in water treatment options [Centers for Disease Control and Prevention (CDC) 2004]. The report includes results from a longitudinal analysis of all childhood blood lead screening results from 1998 to 2003, showing an increase in the percent of BLLs > 5 μg/dL, which coincided with the change to chloramine disinfectant use. Additionally, BLLs were reported from a very limited nonrandom sample (*n* = 201) of residents in 52 households with high (> 300 ppb) lead in drinking water levels. Few conclusions can be drawn from this household data because 53% of those sampled were drinking filtered water (CDC 2004).

Using geographic information system (GIS)-based analysis, we sought to test the potential effect on childhood BLLs of switching to chloramines for disinfection in water treatment systems using data from Wayne County, located in the central Coastal Plain of North Carolina. In particular, we sought to answer three key questions:

- Are changes in BLLs detectable after switching from chlorines to chloramines for disinfection in water treatment systems?
- How do these changes differ according to the age of housing where the child resides?
- How can the answers to the first two questions help guide policy practice?

GISs have many applications in public and environmental health (Miranda et al. 2005; Vine et al. 1997) and have been well applied to research on lead exposure risk (Krieger et al. 2003; Miranda et al. 2002; Reissman et al. 2001; Roberts et al. 2003). GIS allows for the observation and analysis of complex spatio-temporal patterns that may be otherwise overlooked in traditional research and surveillance (Rushton 2003). Spatially based analysis is thus especially well suited to help determine whether changes in water treatment systems introduce systematic changes in childhood BLLs.

Wayne County provides an ideal setting for evaluating these questions for several reasons. First, the housing stock is distributed across a wide variety of age classes (Table 1), with approximately 15.6% built before 1926, 9.3% between 1926 and 1950, 35.5% between 1951 and 1975, and 39.6% after 1975. Second, Wayne County screens a relatively large proportion of 1- and 2-year-old children for lead, ranging from 75.5% in 2000 to 76.1% in 2003. As shown in Table 1, the children screened for lead are well-distributed across the housing age classes in the county. Third, Wayne County contains two main public water systems that together provide water for approximately three-fourths of the residential tax parcels within the county. Approximately 70% of residential tax parcels obtain drinking water through the Wayne Water Systems (WWS). These systems use chlorine for disinfection and sodium fluoride for fluoridation, and do not use an anticorrosive; these treatment options did not change over the course of the study period (1999–2003). Another 28% of residential tax parcels obtain drinking water through the Goldsboro Water System (GWS). This system uses fluorosilicic acid for fluoridation and zinc orthophosphate for anticorrosion. The GWS switched from using chlorine to using chloramines for disinfection in March 2000. This combination of sources of drinking water and treatment strategies allow us to compare outcomes within and across water systems. Figure 1 shows the geographic coverage for each of the water treatment systems across Wayne County.

## Materials and Methods

To analyze potential effects on BLLs associated with changes in water treatment processes, we first built a unified GIS consisting of tax parcel, water treatment system boundary, census, and geocoded blood lead surveillance data. The tax parcel data obtained from the Wayne County GIS Department in 2005 contained information on the year the house was built, owner name and address, sale price and date, heated square feet, and physical address of residential tax parcels. The attribute data within the tax parcel dataset were used to geocode blood lead surveillance data, as well as to determine the year built for houses where screened children resided. Potable service districts data were downloaded from the Wayne County GIS spatial data explorer (Wayne County GIS Department 2006). This dataset contains basic data for each of the water systems within Wayne County, including district name, number, and land area.

Blood lead surveillance data were obtained from the North Carolina Childhood Lead Poisoning Prevention Program, which maintains a statewide registry of all blood lead screens conducted on North Carolina children under the age of 6 years. These data include 18,284 records for Wayne County (11,556 children) with test date, test result, child’s name, and child’s home address. The latter two fields were used to geocode the 1999–2003 surveillance data to the tax parcel data allowing us to create a GIS with 13,231 geocoded records (8,607 children). This GIS linked the blood lead screening data with age of housing, drinking water source, and census data for 7,270 records (U.S. Census Bureau 2000). For children who were screened more than once during this time period, we selected the record with the highest reported BLL for each child at each parcel with the earliest screening date. We were able to successfully geocode 72.4% of records (74.5% of children) in the surveillance data for 1999–2003, with many of the nongeocoded records in the rural areas of the county, in mobile home parks, or receiving mail at a post office box.

We analyzed the data using both exploratory methods and more formal multivariate techniques. Because the distribution of blood lead screens is skewed toward the origin, we used the natural logarithm of the BLL as our dependent variable. In addition, because some residential parcels had more than one linked blood lead screen (e.g., different tenants/owners or multiple siblings from the same family), we used cluster analysis to ensure that we properly weighted the contribution of any one residence to the analysis. We classified each blood lead screen according to whether the drinking water source used chloramines in the disinfection process with a binary (0/1) variable (Table 2).

Previous research indicates that BLLs are typically related to the year the child’s residence was built and demographic variables such as household income and percent African-American race (Lanphear et al. 1998; Miranda et al. 2002; Pirkle et al. 1998; Roberts et al. 2003; Sargent et al. 1995); therefore, we included these variables in the analysis as well. Our general approach for specifying a parcel level model follows the methods established previously (Miranda et al. 2002).

## Results

**Figure 2:**
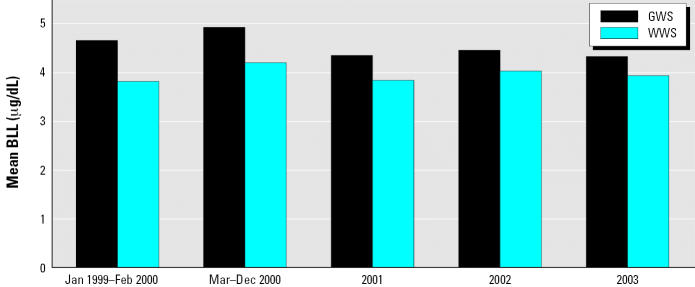
Mean BLLs over time for each drinking water source. The GWS started using chloramines in March 2000.

Exploratory graphical analysis reveals some interesting relationships. Figure 2 shows mean BLLs over time differentiated by drinking water source (WWS or GWS) and highlights the point in time when the GWS switched to chloramines for water disinfection. For both sources of drinking water, mean BLLs increased between the January 1999–February 2000 and the March 2000–December 2000 time periods.

A two sample *t*-test assuming equal variances revealed that the mean BLL was significantly higher (*p* < 0.00001) for children residing in residential tax parcels whose water source relied on chloramines for disinfection (mean BLL = 4.93 μg/dL) compared with those whose water source did not rely on chloramines (mean BLL = 4.19 μg/dL). This may, of course, result solely from artifacts such as the GWS serving more of the older housing stock or the GWS serving more homes in less well-maintained areas.

**Figure 3:**
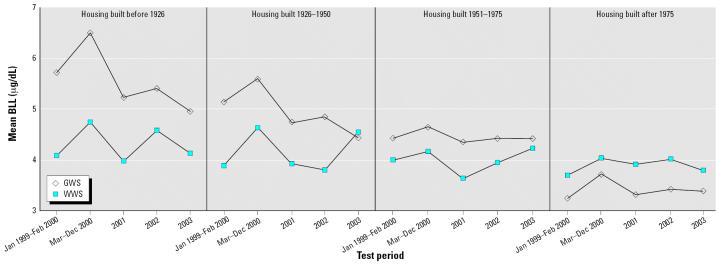
Mean BLLs by test period, by age of housing.

Exploratory graphical analysis was designed to look at differences in BLLs across age classes of homes. Figure 3 shows mean BLL by test year, differentiating between GWS and WWS. These figures are broken out by age of housing categories (pre-1926pre-1926–1950–1951–1975, after 1975). The mean BLLs are virtually indistinguishable across the different water treatment systems for housing built after 1950. This is consistent with empirical observations that older age classes of homes are more likely to contain lead pipes or lead-containing fixtures or solder (Safe Drinking Water Act Amendments of 1986; Troesken and Beeson 2003; U.S. EPA 1991).

Although the exploratory analysis provides insights regarding variables of interest, the question of whether the switch to chloramines by the GWS affected BLLs in children must be explored using multivariate analysis. We used log-linear cluster analysis of blood lead screens, with explanatory variables that included year built; census measures of income and percent African American; three dummy variables for seasons when the blood samples were taken (winter as reference) to control for any potential effect of seasonal variation in BLLs; a dummy variable indicating whether chloramines were being used by the water treatment system that served the home residence of the child at the time of the blood lead screen; and interactive terms that combined year built with the chloramine-use variable. The interactive terms were incorporated in two ways. First, we constructed an interactive variable (year built × chloramines) that allows the effect of chloramines to be mitigated or exacerbated (we anticipated that it should be mitigated) with each incremental year added to year built; that is, we expected that the effect of chloramines on BLL would be less important and eventually unimportant as we moved into newer and newer housing stock. As shown in Table 3, this was borne out in the analysis. All coefficients on demographic and seasonal covariates were of the expected sign and consistent with previous studies.

These results indicate that the change to chloramine disinfection led to an increase in BLLs, the impact of which is progressively mitigated in newer housing. This makes sense in that increases in dissolved lead in water can only happen when a lead source is present, a condition that is much more likely in older housing. The year of construction at which the newness of the housing exerts a stronger influence on BLLs than use of chloramines is 1951, as calculated from the model shown in Table 3. Data on the age of housing contain inaccuracies, especially for the oldest housing stock. Because of these inaccuracies, the model presented in Table 3 (which uses a continuous age-of-housing variable) is vulnerable to bias in estimating the year built coefficient.

To avoid this bias, we also estimated a model using categorical age-of-housing variables. Given the differences across age classes demonstrated in Figure 3 and the crossing point calculated from the model presented in Table 3, we also chose to construct an interactive term that split housing age categories into 25-year increments. Thus we included (year built before 1926 × chloramines), (year built 1926–1950 × chloramines), (year built 1951–1975 × chloramines), and (year built after 1975 × chloramines) as explanatory variables, with the newest housing group serving as the referent group. These results are shown in Table 4.

Again, all coefficients on demographic and seasonal covariates were of the expected sign and of very similar size to the same coefficients in Table 3. These results also demonstrate a dose–response effect vis-à-vis age of housing; that is, the increase in BLLs for children screened at locations or times where chloramines were used is greatest in houses built before 1926, followed by houses built in 1926–1950, followed by houses built in 1951–1975.

## Discussion

When municipal water treatment systems introduce the use of chloramines for the purpose of reducing carcinogenic by-products, they may inadvertently increase exposure to lead in the water supply. Tables 3 and 4 indicate that use of chloramines in Wayne County, North Carolina, is a significant predictor of BLLs, and the extent of the effect decreases in newer housing stock. Definitive conclusions regarding the use of chloramines are difficult because of the particular combinations of disinfection agents, anticorrosives, coagulants, and fluoride additives used in water treatment systems. We are currently pursuing the data to undertake a more comprehensive analysis.

Although we were not able to perform a more expansive analysis at this time, the results presented in this article provide policy guidance to municipalities. In prioritizing children to screen for elevated BLLs when chloramines are not being used, local health departments should target children living in housing built before 1950. Similarly, when chloramines are being used, local health departments should target children living in housing built before 1975. This suggests that, assuming the results from Wayne County can be generalized to other areas, local health departments need to expand their scope of targeted screening in the years following the introduction of chloramines as a water-disinfection agent. Local health department officials may also wish to provide more intensive outreach and education to residents of older housing, including techniques such as flushing water pipes before consumption.

In addition, after changes in water treatment, surveillance on lead in water can focus on housing built before 1975, in spite of the fact that lead solder was not banned until 1986. Additional analyses indicate that after introducing chloramines, again assuming the Wayne County case can be generalized, local health departments can focus both blood lead screen and water-testing efforts on that part of the housing stock built before 1965 (Miranda ML, Kim D, Hull AP, Paul CJ, Overstreet Galeano MA, unpublished data). Because routine monitoring failed to detect increases in water lead levels both in North Carolina and in Washington, DC, we wish to emphasize the recommendations of water chemistry experts Edwards and Dudi (2004) and Schock (1990): *a*) water lead levels should be closely monitored after changing to use of chloramines; and *b*) a sampling program designed to fully document the variability of water lead levels should be implemented. Appropriate and timely collection of water lead level data would enable meaningful assessment of human exposure and also provide water treatment systems with insights regarding which exposure control strategies are most appropriate.

## Conclusions

Several caveats must be mentioned that temper the possible conclusions from this study. First, although the results are directly relevant to Wayne County, the extent to which they can be extrapolated to other areas in North Carolina, the Southeastern United States, or the United States more generally is yet to be determined. We are currently pursuing the data required to replicate the current study in other locations. Second, blood lead surveillance data are decidedly nonrandom in that programs typically target children living in the highest risk housing, based, in many cases, on age of housing. However, because Wayne County screens such a high proportion of young children, this concern is somewhat mitigated. In spite of targeted screening efforts, the distribution of age of housing where sampled children resided differs by < 2 percentage points from the distribution for the whole county. Third, some environmental chemists hypothesize that the dissolution of lead from pipes into water after switching to chloramines is a transient process, because a new coating may eventually develop on the inside of pipes, effectively creating a new barrier between the water and the lead source. This temporal dimension is unexplored in the current analysis. Fourth, we did not analyze lead in water directly and thus can only indirectly suggest that the increase in BLLs after the switch to chloramines was caused by an increase in lead in drinking water. We are currently working with the State of North Carolina to identify houses to sample for lead in water based on a geographic sampling design; we will analyze these data when they become available.

In the present study we directly analyzed the effects of changes in water treatment options on BLLs. This study provides important policy guidance to communities that may be balancing trihalomethane exceedances against possible increases in lead in drinking water. As similar research is conducted in other areas, and the underlying environmental chemistry is clarified, water treatment strategies can be optimized across the multiple objectives that municipalities face in providing high-quality drinking water to local residents.

## Figures and Tables

**Figure 1:**
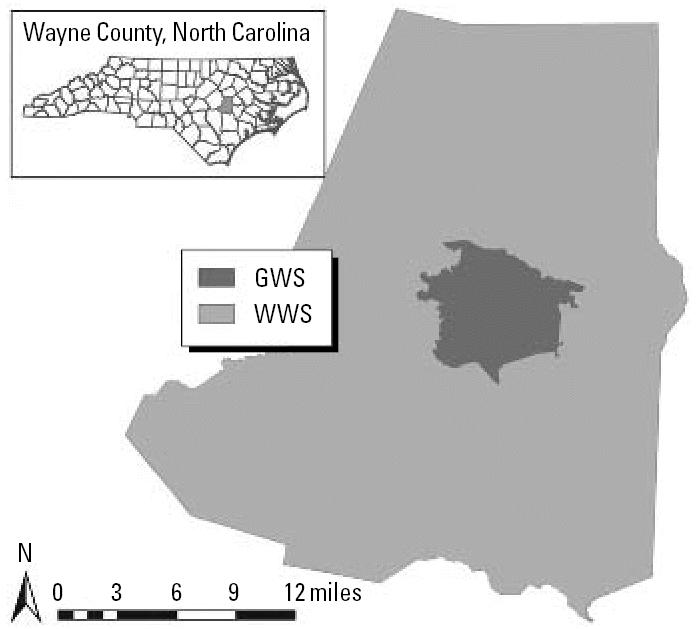
Wayne County potable water systems.

**Table 1:** Distribution of year built for housing stock and for residences of screened children.

Year built (residential)	Residences of screened children (%)	Wayne County housing stock (%)
Pre-1926	16.5	15.6
1926–1950	7.7	9.3
1951–1975	36.0	35.5
After 1975	39.8	39.6

**Table 2:** Use of chloramines by drinking water source for geocoded blood lead surveillance data.

	Chloramine use (no. of screens)	
Period	WWS	GWS
Jan 1999–Feb 2000	No (849)	No (651)
Mar 2000–Dec 2003	No (3,215)	Yes (2,555)

**Table 3:** Clustered multivariate regression results using a simple interaction term.

	Dependent variable: ln (BLL)		
Variable	Coefficient	SE	*P* > |*t*|
Year built (continuous)	−1.55 × 10^−3^	3.74 × 10^−4^	0.000
Household median income	−4.98 × 10^−6^	1.02 × 10^−6^	0.000
Percent African American	1.60 × 10^−3^	3.14 × 10^−4^	0.000
Use of chloramines	4.659	1.32	0.000
Use of chloramines × year built	−2.38 × 10^−3^	6.74 × 10^−4^	0.000
Screened in spring	−0.020	0.025	0.427
Screened in summer	0.078	0.025	0.001
Screened in fall	0.083	0.025	0.001
Constant	4.395	0.730	0.000

The referent group for the season variables is winter (December, January, and February).

**Table 4:** Clustered multivariate regression results using categorical age of housing interaction term.

	Dependent variable: ln (BLL)		
Variable	Coefficient	SE	*P* > |*t*|
Year built before 1926	0.166	0.356	0.000
Year built 1926–1950	0.120	0.049	0.015
Year built 1951–1975	0.005	0.025	0.841
Household median income	−4.93 × 10^−6^	1.03 × 10^−6^	0.000
Percent African American	1.66 × 10^−3^	3.18 × 10^−4^	0.000
Use of chloramines	−0.087	0.038	0.021
Use of chloramines × year built before 1926	0.167	0.062	0.008
Use of chloramines × year built 1926–1950	0.161	0.071	0.024
Use of chloramines × year built 1951–1975	0.108	0.048	0.023
Screened in spring	−0.022	0.025	0.376
Screened in summer	0.078	0.025	0.002
Screened in fall	0.081	0.025	0.001
Constant	1.327	0.0466	0.000

The referent group for the interaction term is use of chloramines × year built after 1975.
